# Supplementary material for: Impact of a Moderately Hypocaloric Mediterranean Diet on the Gut Microbiota Composition of Italian Obese Patients
Source: Nutrients. 2020 Sep 4;12(9):2707. doi: 10.3390/nu12092707 (PMC7551852; doi:10.3390/nu12092707)
Supplement: Supplementary file 1 [file nutrients-12-02707-s001.pdf]

Table S1. Differences in the anthropometric data before and after the nutritional intervention (NI) by BMI categories, evaluated by one-way ANOVA with repeated measures.

Anthropometric variable before and after the NI		Overweight (N= 3), M(SD)	Obesity class I (N=8), M(SD)	Obesity class II (N=9), M(SD)	Obesity class III (N=3), M(SD)
BW (Kg)	Before NI	67.1 (2.2)	78.9 (7.3)	93.5 (8.1)	128.0 (12.9)
	After NI	63.3 (2.4)	74. 2 (9.1)	89.3 (9.7)	116.7 (7.3)
	Direction	↓	↓	↓	↓
	p-value	0.052	0.048	0.265	0.369
FM (Kg)	Before NI	23.3 (2.8)	34.0 (4.6)	41.0 (3.6)	52.8 (16.0)
	After NI	20.5 (3.9)	29.6 (5.3)	37.1 (3.9)	44.4 (6.4)
	Direction	↓	↓	↓	↓
	p-value	0.057	0.054	0.001	0.474
MM (Kg)	Before NI	41.6 (1.4)	38.0 (14.0)	49.0 (3.9)	71.7 (5.0)
	After NI	40.6 (1.4)	42.5 (3.7)	49.7 (6.3)	68.7 (0.8)
	Direction	↓	↑	↑	↓
	p-value	0.175	0.723	0.615	0.916
WC (cm)	Before NI	85.0 (4.6)	102.0 (7.1)	114.1 (5.8)	129.0 (6.6)
	After NI	81.0 (7.3)	98.5 (7.6)	107. 4 (14.0)	123.5 (3.5)
	Direction	↓	↓	↓	↓
	p-value	0.424	0.054	0.640	0.430

BW = bodyweight, FM = fat-mass, M = Mean, MM = muscle mass, SD = Standard deviation, WC = waist circumference.

Table S2. Differences in the means of Shannon index before and after the nutritional intervention (NI) by BMI categories, evaluated by one-way ANOVA with repeated measures. We observed a significant increase in alpha diversity in patients with class I obesity. No statistically significant results were obtained in all the other categories.

BMI category	M (SD) before NI	M (SD) after NI	Direction	p
Overweight (N=3)	3.50 (0.56)	3.49 (0.41)	↓	0.497
Obesity class I (N=8)	3.49 (0.33)	3.52 (0.33)	↑	0.007
Obesity class II (N=9)	3.17 (0.69)	3.10 (0.88)	↓	0.323
Obesity class III (N=3)	3.84 (0.29)	3.87 (0.61)	↑	0.508

M = Mean, SD = Standard deviation.

Table S3. Comparison of the Bray-Curtis distance between different BMI categories, evaluated by Permutational Multivariate Analysis of Variance (PERMANOVA). No statistically significant results were obtained both at baseline and after the nutritional intervention (NI).

PERMANOVA results before NI	PERMANOVA results after NI
p = 0.796	p = 0.090
R^2 = 0.105	R^2 = 0.187
F = 0.743	F = 1.457

Table S4. Comparison of the Bray-Curtis distance between males and females, evaluated by Permutational Multivariate Analysis of Variance (PERMANOVA). No statistically significant results were obtained both at baseline and after the nutritional intervention (NI).

PERMANOVA results before NI	PERMANOVA results after NI
p = 0.539	p = 0.649
$R^2 = 0.039$	$R^2 = 0.031$
F = 0.865	F = 0.661

Table S5. Differences in the ratio Firmicutes/Bacteroidetes before and after the nutritional intervention (NI) by BMI categories, evaluated by one-way ANOVA with repeated measures. No statistically significant results were obtained in all the different categories.

BMI category	M (SD) before NI	M (SD) after NI	Direction	p
Overweight (N=3)	3.19 (2.05)	2.10 (2.10)	↓	0.699
Obesity class I (N=8)	7.68 (5.62)	3.21 (4.46)	↓	0.109
Obesity class II (N=9)	3.72 (5.60)	2.09 (1.64)	↓	0.457
Obesity class III (N=3)	1.46 (1.01)	1.64 (1.18)	↑	0.791

M = Mean, SD = Standard deviation

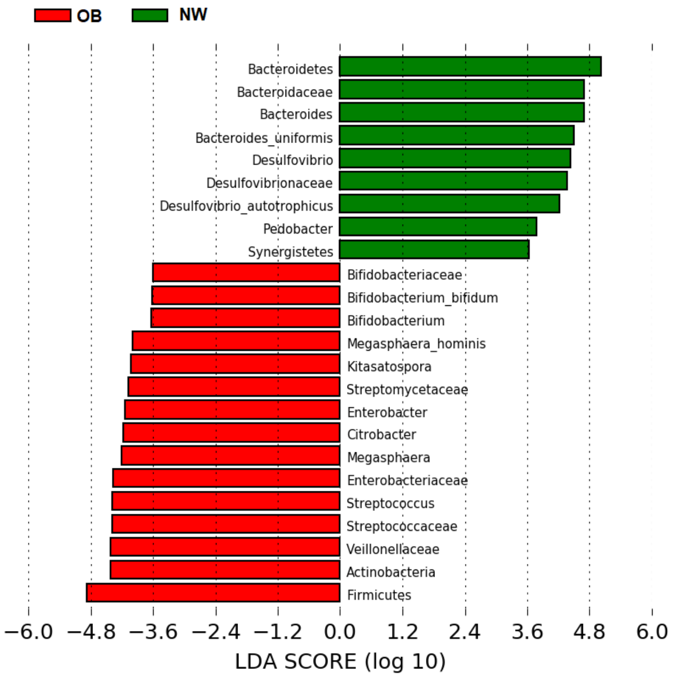

Figure S1. LEfSe algorithm evaluating the differences in bacterial relative abundance between obese and overweight patients (OB) and normal-weight healthy controls (NW). Analysis restricted to female patients (N = 20). Results are ranked by the Linear Discriminant Analysis value (LDA score): bacteria in red were more expressed abundant in OB, while bacteria in green were more abundant in NW. All the results were confirmed after FDR adjustment.

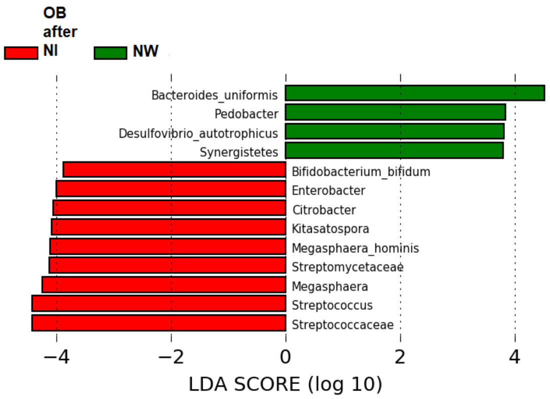

Figure S2. LEfSe algorithm evaluating the differences in bacterial relative abundance between obese and overweight patients (OB) after the nutritional intervention (NI) and normal-weight healthy controls (NW). Analysis restricted to female patients (N = 20). Results are ranked by the Linear Discriminant Analysis value (LDA score): bacteria in red were more expressed abundant in OB, while bacteria in green were more abundant in NW. All the results were confirmed after FDR adjustment.

Table S6. Significant changes in the bacterial relative abundance after the nutritional intervention in female patients (N = 20).

**Phylum**	**Family**	***Genus***	***Species***	**Median (IQR) at T0**	**Median (IQR) at T3**	**Prevalent direction of change (N)**	**p**	**q**
Bacteroidetes	Bacteroidaceae	*Bacteroides*	*B. cellulosyliticus*	0.019 (0.060)	0.042 (0.300)	↑ (17)	0.012	0.266
			B. rodentium	0.764 (0.858)	1.461 (2.577)	↑ (14)	0.023	0.278
			B. uniformis	0.827 (1.011)	2.017 (2.640)	↑ (14)	0.015	0.266
	Paraprevotellaceae			0.186 (0.432)	0.472 (1.192)	↑ (15)	0.037	0.347
	Porphyromonadaceae	*Parabacteroides*		0.725 (0.607)	1.427 (2.054)	↑ (14)	0.030	0.325
			P. distasonis	0.253 (0.351)	0.394 (0.646)	↑ (15)	0.030	0.325
	Sphingobacteriaceae	*Sphingobacterium*	*S. shayense*	0.041 (0.045)	0.077 (0.089)	↑ (15)	0.023	0.278
	Prevotellaceae	*Prevotella*	*P. stercorea*	0 (0)	0 (0.001)	≡ (13), ↑ (7)	0.018	0.266
Firmicutes	Acidaminococcaceae	*Acidaminococcus*	*A. fermentans*	0.003 (0.010)	0.018 (0.029)	↑ (14)	0.037	0.347
		Dialister	*D. invisus*	0.002 (0.003)	0.003 (0.010)	↓ (15)	0.048	0.406
	Erysipelotricaceae	*Catenibacterium*		0.002 (0.102)	0.005 (0.180)	↑ (16)	0.005	0.266
	Lachnospiraceae	*Coprococcus*	*C. eutactus*	0.003 (0.062)	0.016 (0.371)	↑ (18)	0.002	0.266
		Dorea		0.379 (0.639)	0.112 (0.514)	↓ (15)	0.040	0.361
		Roseburia	*R. faecis*	0.437 (0.773)	0.283 (0.374)	↓ (15)	0.030	0.325
	Selenomonadaceae	*Megamonas*		0 (0.001)	0 (0)	≡ (12), ↓ (7)	0.017	0.266
			M. funiformis	0 (0.001)	0 (0)	≡ (12), ↓ (8)	0.012	0.266
	Streptococcaceae			0.233 (0.376)	0.140 (0.145)	↓ (14)	0.015	0.266
		Streptococcus		0.222 (0.383)	0.114 (0.146)	↓ (14)	0.015	0.266
		Streptococcus	*S. vestibularis*	0.042 (0.064)	0.013 (0.035)	↓ (17)	0.008	0.266
	Veillonellaceae	*Veillonella*		0.057 (0.118)	0.151 (0.266)	↑ (16)	0.015	0.266

Median = median values of the bacterial relative abundance before (T0) and after (T3) NI (bacterial relative abundance is expressed as percentage from 0 to 100); IQR = interquartile range calculated as the difference between upper and lower quartiles; N = number of patients that reported the most prevalent direction of change in the bacterial relative abundance (↓ = reduced after NI, ↑ = increased after NI, ≡ = no different after NI). Results were obtained by the Wilcoxon test for paired data performed on R software (v. 3.5.2). q = p adjusted for Benjamini and Hochberg’s false discovery rate (FDR) correction test for multiple comparisons.

nutrients

MDPI

Table S7. Metadata of the participants included in the study. Overweight and obese patients (OB) = 23, Normal-weight controls (NW) = 46.

Id	*Group*	*Sex*	*Age*	*Waist circumference (cm)*	*FM (%)*	*FM (kg)*	*MM (kg)*	*BMR (kcal)*	*BMI*	*Daily caloric intake (Kcal)*	*Carbohydrates intake %*	*Lipids intake %*	*Saturated lipids intake/ [illegible]*	*Daily proteins intake (gr)*	*Daily fiber intake (gr)*	*MedDietScore*	*Current Smoking status*	*Former Smoking status*	*Current Alcohol consumption*	*Hypertension*	*Dyslipidemia*	*Insuline resistence*	*Type II diabetes*
N002F	OB	Female	42	102	42.1	36.2	47.4	1538	31.6	2271	40	38	38	125	6	19	Yes	No	Rare	No	No	No	No
N004F	OB	Female	66	115	40.7	33.9	46.8	1491	39.0	1680	47	31	29	92	18	33	No	No	None	Yes	No	No	No
N005F	OB	Female	57	86	35.8	24.8	42.3	1340	26.8	1396	52	27	28	71	27	33	Yes	No	Rare	No	No	No	No
N006F	OB	Male	45	126	39.9	44.7	64.0	2054	38.8	2962	43	39	38	133	15	20	Yes	No	Rare	Yes	No	Yes	No
N007F	OB	Female	53	135	50.2	71.0	66.9	2231	40.9	2188	53	30	39	86	12	31	No	No	None	Yes	No	No	No
N008F	OB	Female	61	89	37.3	25.0	40.0	1274	29.8	2694	41	34	40	64	17	23	No	No	None	Yes	No	No	No
N009F	OB	Female	45	110	44.1	39.1	47.1	1537	39.4	1921	49	36	36	77	19	30	No	No	Rare	No	No	No	No
N011F	OB	Male	40	122	35.3	40.8	71.1	2273	40.5	2572	56	31	42	86	19	27	Yes	No	Moderate	No	No	No	No
N012F	OB	Female	50	113	44.8	38.5	45.1	1474	34.0	1964	49	37	40	67	9	24	No	Yes	Moderate	No	No	No	No
N014F	OB	Female	49	111	46.3	44.5	49.1	1609	37.6	1470	43	41	39	64	16	29	No	No	None	No	No	No	No
N016F	OB	Female	55	112	44.1	38.5	46.3	1503	38.3	1025	50	39	33	71	5	30	No	Yes	Moderate	No	No	No	No
N020F	OB	Female	57	98	42.6	35.2	45.0	1452	31.1	1483	41	44	45	52	9	30	No	No	Rare	No	No	No	No
N022F	OB	Female	48	106	45.2	42.7	49.2	1606	35.1	1908	49	32	45	86	17	35	No	No	None	No	No	Yes	No
N023F	OB	Male	49	130	36.5	46.6	77.0	2476	42.6	1530	53	29	36	73	18	31	No	No	None	Yes	Yes	Yes	No
N024F	OB	Female	52	112	46.5	44.2	40.2	1580	37.2	1515	61	26	43	51	15	36	Yes	No	Moderate	No	No	No	No
N026F	OB	Female	58	99	45.3	36.6	4.0	1376	33.2	1052	45	35	36	59	14	27	No	No	None	No	Yes	No	No
N027F	OB	Female	59	94	40.5	27.0	37.6	1220	31.7	751	61	24	48	29	6	30	Yes	No	None	No	No	No	No
N028F	OB	Female	67	116	42.2	38.7	50.4	1611	36.8	1870	56	24	39	71	16	35	No	No	Rare	No	Yes	No	Yes
N029F	OB	Female	32	80	31.0	20.0	42.6	1366	27.0	1625	43	38	49	74	9	27	No	No	Rare	No	No	No	No
N035F	OB	Female	56	108	44.3	36.3	43.3	1410	33.7	2076	56	33	32	57	24	28	No	Yes	None	No	Yes	No	No
N037F	OB	Female	62	98	40.6	27.9	38.8	1254	34.6	1821	49	37	38	62	12	37	No	Yes	Rare	No	Yes	No	No
N039F	OB	Female	67	96	40.1	31.9	45.2	1438	33.5	1779		33	40	71	14	37	No	Yes	Moderate	Yes	Yes	No	No
N044F	OB	Female	53	120	45.5	42.3	48.1	1570	35.9	1358	59	27	37	49	5	26	No	No	None	Yes	No	No	Yes
BD001	NW	Male	58	87	NA	NA	NA	NA	24.2	1825	52	23	25	80	18	43	No	No	None	No	No	No	No
BD002	NW	Female	50	79	NA	NA	NA	NA	24.4	1433	52	25	31	56	20	35	No	No	Rare	No	No	No	No

Id	*Group*	*Sex*	*Age*	*Waist circumference (cm)*	*FM (%)*	*FM (kg)*	*MM (kg)*	*BMR (kcal)*	*BMI*	*Daily caloric intake (Kcal)*	*Carbohydrates intake %*	*Lipids intake %*	*Saturated lipids intake/ [illegible]*	*Daily proteins intake (gr)*	*Daily fiber intake (gr)*	*MedDietScore*	*Current Smoking status*	*Former Smoking status*	*Current Alcohol consumption*	*Hypertension*	*Dyslipidemia*	*Insuline resistence*	*Type II diabetes*
BD003	NW	Female	29	75	NA	NA	NA	NA	23.7	1378	49	26	32	54	22	30	No	No	None	No	No	No	No
BD005	NW	Female	60	72	NA	NA	NA	NA	21.6	1410	54	31	34	50	23	37	No	No	Rare	No	No	No	No
BD006	NW	Female	58	70	NA	NA	NA	NA	19.1	1289	55	30	32	60	21	31	No	No	None	No	No	No	No
BD009	NW	Female	45	72	NA	NA	NA	NA	20.7	1324	53	22	31	63	24	24	No	No	Moderate	No	No	No	No
BD011	NW	Female	39	71	NA	NA	NA	NA	18.9	1267	47	27	30	67	17	36	No	No	Rare	No	No	No	No
BD012	NW	Male	27	80	NA	NA	NA	NA	19.3	1621	50	25	27	66	16	38	No	No	Moderate	No	No	No	No
BD017	NW	Female	25	70	NA	NA	NA	NA	19	1267	49	29	29	57	15	31	Yes	No	None	No	No	No	No
BD019	NW	Female	29	73	NA	NA	NA	NA	19.2	1321	50	20	28	54	25	28	No	No	Moderate	No	No	No	No
BD020	NW	Male	53	86	NA	NA	NA	NA	23.7	1780	48	25	31	78	19	33	No	No	Rare	No	No	No	No
BD026	NW	Female	30	72	NA	NA	NA	NA	21.4	1410	52	20	29	52	20	39	No	No	Moderate	No	No	No	No
BD028	NW	Female	45	70	NA	NA	NA	NA	20.6	1423	54	19	20	56	23	35	No	No	Rare	No	No	No	No
BD029	NW	Female	24	64	NA	NA	NA	NA	19.1	1334	49	27	22	53	21	35	Yes	No	Moderate	No	No	No	No
BD032	NW	Female	63	76	NA	NA	NA	NA	23.4	1523	47	25	26	51	17	35	No	No	Rare	No	No	No	No
BD039	NW	Female	33	68	NA	NA	NA	NA	19.2	1323	56	30	29	59	17	33	No	No	Moderate	No	No	No	No
BD041	NW	Male	44	81	NA	NA	NA	NA	21	1690	53	30	21	75	18	37	No	No	Rare	No	No	No	No
BD042	NW	Female	45	74	NA	NA	NA	NA	20.3	1434	52	23	22	61	17	29	No	No	Rare	No	No	No	No
BD044	NW	Male	52	87	NA	NA	NA	NA	23.8	1850	54	16	34	54	18	38	Yes	No	Rare	No	No	No	No
BD049	NW	Female	52	70	NA	NA	NA	NA	20.3	1345	47	20	27	57	24	31	No	No	None	No	No	No	No
BD050	NW	Male	39	85	NA	NA	NA	NA	24.2	1820	49	21	26	82	21	38	Yes	No	None	No	No	No	No
BD061	NW	Female	57	73	NA	NA	NA	NA	22.8	1435	50	22	33	68	18	33	No	No	Rare	No	No	No	No
BD062	NW	Female	48	75	NA	NA	NA	NA	22.6	1453	51	23	30	59	16	32	No	No	Rare	No	No	No	No
BD063	NW	Female	55	72	NA	NA	NA	NA	22.3	1421	55	30	31	63	23	34	No	No	Moderate	No	No	No	No
BD064	NW	Female	60	71	NA	NA	NA	NA	21.8	1390	49	28	24	60	22	30	No	No	Moderate	No	No	No	No
BD066	NW	Female	59	72	NA	NA	NA	NA	23.3	1456	48	31	28	55	20	34	Yes	No	Moderate	No	No	No	No
BD067	NW	Female	46	76	NA	NA	NA	NA	20.1	1324	50	29	27	63	21	44	No	No	Rare	No	No	No	No
BD069	NW	Female	56	71	NA	NA	NA	NA	21.5	1423	55	33	26	86	22	32	No	No	None	No	No	No	No

Id	*Group*	*Sex*	*Age*	*Waist circumference (cm)*	*FM (%)*	*FM (kg)*	*MM (kg)*	*BMR (kcal)*	*BMI*	*Daily caloric intake (Kcal)*	*Carbohydrates intake %*	*Lipids intake %*	*Saturated lipids intake/ [illegible]*	*Daily proteins intake (gr)*	*Daily fiber intake (gr)*	*MedDietScore*	*Current Smoking status*	*Former Smoking status*	*Current Alcohol consumption*	*Hypertension*	*Dyslipidemia*	*Insuline resistence*	*Type II diabetes*
BD070	NW	Female	53	72	NA	NA	NA	NA	23.3	1569	53	31	23	65	23	30	No	No	None	No	No	No	No
BD071	NW	Female	62	73	NA	NA	NA	NA	20.28	1390	52	30	13	64	19	34	No	No	None	No	No	No	No
BD072	NW	Female	56	66	NA	NA	NA	NA	22.2	1410	47	29	21	86	18	30	No	No	Rare	No	No	No	No
BD073	NW	Female	53	69	NA	NA	NA	NA	19.92	1321	52	27	33	60	25	36	No	No	Rare	No	No	No	No
BD074	NW	Female	54	79	NA	NA	NA	NA	24.81	1678	48	30	32	64	19	35	No	No	Rare	No	No	No	No
BD076	NW	Female	56	75	NA	NA	NA	NA	22.14	1543	52	31	31	57	17	30	No	No	Rare	No	No	No	No
BD077	NW	Female	52	74	NA	NA	NA	NA	23.3	1567	51	29	30	62	26	29	No	No	Rare	No	No	No	No
BD078	NW	Female	57	76	NA	NA	NA	NA	24	1534	55	30	29	64	24	36	No	No	None	No	No	No	No
BD079	NW	Female	52	69	NA	NA	NA	NA	19.2	1304	51	27	27	58	19	29	No	No	Moderate	No	No	No	No
BD080	NW	Female	50	79	NA	NA	NA	NA	24.6	1678	48	34	26	59	10	28	Yes	No	Moderate	No	No	No	No
BD081	NW	Female	60	74	NA	NA	NA	NA	23.2	1567	46	29	30	59	18	30	No	No	Rare	No	No	No	No
BD082	NW	Female	49	69	NA	NA	NA	NA	20.8	1308	57	31	29	64	17	34	Yes	No	Rare	No	No	No	No
BD083	NW	Female	50	78	NA	NA	NA	NA	24.6	1657	45	28	31	65	22	29	No	No	Rare	No	No	No	No
BD084	NW	Female	71	65	NA	NA	NA	NA	18.1	1278	53	32	25	54	19	40	Yes	No	Moderate	No	No	No	No
BD085	NW	Female	43	68	NA	NA	NA	NA	20	1480	49	30	23	57	24	36	No	No	Rare	No	No	No	No
BD086	NW	Female	53	72	NA	NA	NA	NA	20.7	1400	52	29	34	62	23	34	No	No	Rare	No	No	No	No
BD088	NW	Female	56	63	NA	NA	NA	NA	17.2	1256	55	34	27	68	20	32	No	No	Rare	No	No	No	No
BD089	NW	Female	52	78	NA	NA	NA	NA	24.5	1610	47	32	29	59	21	30	No	No	Rare	No	No	No	No
